# Evaluation of antigenicity and nutritional properties of enzymatically hydrolyzed cow milk

**DOI:** 10.1038/s41598-021-98136-z

**Published:** 2021-09-20

**Authors:** Xiaona Liang, Guanlin Qian, Jing Sun, Mei Yang, Xinyang Shi, Hui Yang, Junrui Wu, Zongzhou Wang, Yan Zheng, Xiqing Yue

**Affiliations:** College of Food Science, Shenyang Agricultural University, Shenyang, 100866 People’s Republic of China

**Keywords:** Biological techniques, Immunology, Chemistry, Engineering

## Abstract

While enzymatic hydrolysis is an effective method for lowering the antigenicity of cow milk (CM), research regarding the antigenicity and nutritional traits of CM hydrolysate is limited. Here, we evaluated the protein content, amino acid composition, sensory traits, color, flow behavior, and antigenicity of CM following enzymatic hydrolysis. The results showed that enzymatic hydrolysis increased the degree of hydrolysis, destroyed allergenic proteins, including casein, β-lactoglobulin, and ɑ-lactalbumin, and significantly increased the content of free amino acids and nutritional quality. In particular, the antigenicity of CM was significantly reduced from 44.05 to 86.55% (*P* < 0.5). Simultaneously, the taste, color, and flow behavior of CM were altered, the sweetness and richness intensity decreased significantly (*P* < 0.5), and astringency and bitterness were produced. A slightly darker and more yellow color was observed in CM hydrolysate. In addition, apparent viscosity decreased and shear stress significantly increased with increasing shear rate intensity. The results will provide a solid theoretical foundation for the development of high-quality hypoallergenic dairy products.

## Introduction

Cow milk (CM) is a typically white or yellow biological fluid with unique components and complexity, and is secreted from the mammary glands of cows. It is an indispensable part of a nutritional diet for infants and children, and it contains a variety of nutrients and bioactive factors, which play important roles in the immune system, growth and development, prevention of virus colonization, and regulation of the intestinal microflora^1^. Moreover, CM is one of the most important foods in the adult diet. However, cow milk allergy (CMA), which affects the digestion and absorption of nutrients in humans and animals, limits the use of CM and CM products.

The incidence of CMA is increasing worldwide^2^. CMA is one of the major causes of food hypersensitivity in infants and children, which might progress into other atopic diseases such as other food allergies, rhinitis, asthma, and early atopic dermatitis^3^. Approximately 2–6% of infants and children exhibit CMA in the first years of life^4^. CMA is closely related to a broad spectrum of IgE-mediated and non-IgE-mediated hypersensitivity disorders. Among them, the clinical signs of CMA involving IgE-mediated reactions are expressed mostly as immediate symptoms, which might affect the skin, respiratory tract, gastrointestinal tract, or systemic anaphylactic shock, such as urticaria and eczema, asthma, rhinoconjunctivitis, vomiting, diarrhea, and colic^5^.

The allergenicity of CM is associated with its protein constituents, of which casein (CN), β-lactoglobulin (BLG), and ɑ-lactalbumin (ALA) are the most prominent allergens recognized by human IgE^6^. Bovine serum albumin (BSA), lactoferrin (LF), immunoglobulins (Igs), and low-content proteins might also be capable of inducing CMA^7^. CMA awareness is a health concern, and the only therapy available is allergen avoidance^8^, which can be achieved through allergen destruction. Thus, developing an effective processing method that can destroy allergens and reduce or eliminate the potential antigenicity of CM is necessary for individuals with CMA.

Currently, many methods to reduce food protein allergenicity are available, including thermal and nonthermal strategies^9^. Among them, thermal treatment is a common strategy to reduce food allergens, but it might destroy the quality and nutritional properties of the food^10^. Nonthermal treatment has recently received more attention, as it can not only effectively reduce the allergenicity but also could retain the nutritional and sensory properties of food. The most popular nonthermal treatments include high-pressure treatment^11^, gamma irradiation^12^, and enzymatic hydrolysis^13^. Gamma irradiation is a useful physical technique for reducing or eliminating the allergenicity of certain milk allergens^14^. Some studies have demonstrated a significant reduction in IgE-binding capacity to BLG with irradiation at 10 kGy^15^. Although irradiation has been applied to the field of food production, it is not well accepted by consumers who are not knowledgeable of this technology. While Lima et al. suggested that high-pressure treatment can lower the antigenicity and allergenicity of food proteins and maintain the original flavor and inherent nutritional quality of food, this treatment method is limited to certain types of food^16^. Enzymatic hydrolysis is a widely accepted and safe technology for reducing the allergenicity of food proteins^17^. During enzymatic hydrolysis, some proteins are destroyed, and the peptide or disulfide bonds are broken, which leads to the collapse of conformational and sequential epitopes, which reduces or eliminates the allergenicity of food^18^. Cabanillas et al. investigated the effects of enzymatic treatment on the immunological reactivity of members of the Leguminosae family in vitro. Results showed a significant decrease in IgE reactivity and Ara h 1, Ara h 2, and Ara h 3 levels in the first 30 min of hydrolyzation with Alcalase^19^. In addition, Duan et al. showed that the allergenicity of whey protein concentrate (WPC) could be significantly reduced by hydrolysis with trypsin^20^.

During the enzymatic hydrolysis treatment, many new peptides are produced, and the nutritional quality, taste, and color of food can be altered^21^. To the best of our knowledge, previous studies have only focused on the immunoreactivity of certain proteins in CM, and information about the effects of enzymatic hydrolysis on the immunoreactivity, nutritional quality, taste, color, and rheological properties of natural CM is rare. In this study, the effects of enzymatic hydrolysis on the immunoreactivity, protein pattern, amino acid (AA) composition, taste, color, and rheological properties of natural CM were systematically evaluated. This work will provide a theoretical foundation for the development of high-quality hypoallergenic dairy products.

## Materials and methods

### Chemicals and reagents

CN (purity > 85%), ALA (purity > 85%), BLG (purity > 85%), o-phthalaldehyde, o-phenylenediamine dihydrochloride, dithiothreitol, and gelatin were purchased from Sigma-Aldrich (St. Louis, MO, USA). Alcalase (catalytic activity: 105,466 U/g), Protamex (catalytic activity: 144,524 U/g), and Flavourzyme (catalytic activity: 20,786 U/g) were obtained from Novozymes (Bagsvaerd, Denmark).

### Polyclonal antibody production

Rabbit serum comprising polyclonal antibodies targeting CM was prepared at laboratory of the Shenyang Agricultural University (Liaoning, China), which according to the method described by Oliveira et al.^22^. All experimental protocols were approved by the Ethical Committee for the Experimental Use of Animals at Shenyang Agricultural University (License No. SYXK < Liao > 2011-0001). All methods were carried out in accordance with the guidelines for the Care and Use of Laboratory Animals published by the U.S. National Institutes of Health (NIH Publication 85-23, 1996). The study followed the recommendations in the ARRIVE guidelines. Briefly, rabbits (New Zealand, male, 6–8 weeks), which were firstly administrated with CM (1 mg dissolved in 0.5 ml saline and 0.5 ml complete Freund’s adjuvant, 0 weeks), and then booster injections (1 mg dissolved in 0.5 ml saline and 0.5 ml incomplete Freund’s adjuvant), which were administrated at 2, 4, 6, 8 weeks. The rabbits were humanely euthanized, which according to the American Veterinary Medical Association (AVMA) Guidelines for the Euthanasia of Animals (2020), the polyclonal antibodies were collected and stored at − 80 °C until further use.

### Sample collection

Skim CM was provided by a local farm in Shenyang (Liaoning, China). CM was collected from 60 healthy Holstein cows (2–5 years of age). The samples were transported to the laboratory and stored at − 80 °C before analysis. The collection of samples was approved by the Shenyang Agricultural University, Chinese Human Research Ethical Committee, and all experiments were performed according to Chinese laws and institutional guidelines.

### Enzymatic hydrolysis

Alcalase, Protamex, and Flavourzyme were diluted to 100 mg/mL with distilled water. The skim CM was preheated for 20 min at the optimal temperature (Alcalase: 55 ± 5 °C; Protamex; 50 ± 5 °C; Flavourzyme: 50 ± 5 °C). Then, followed by the addition of enzyme solutions according to the enzyme activity to substrate ratio (Alcalase, Protamex, and Flavourzyme was 10,000 U/g, 10,000 U/g, and 6,000 U/g, respectively). The mixture solutions were continuously stirred and the reaction was terminated by heating the solution at 90–100 °C for 10 min and cooling on ice water. The solutions were centrifuged at 5000 × *g* for 10 min and stored at − 80 °C until use.

### Determination of the degree of hydrolysis

The degree of hydrolysis (DH) of the hydrolysates was evaluated using the OPA method, according to the method of^23^. In brief, 400 μL of sample was added to 3 mL of OPA solution, the mixture was combined by inversion and incubated for 2 min at room temperature in the dark. A serine solution (100 μg/mL) was used as a standard control. The absorbance was measured at 340 nm using a spectrophotometer.

### Trisine SDS-PAGE

SDS-PAGE was performed according to the procedure described by^24^. Stacking and separating gels were prepared using 3% and 15% acrylamide concentrations, respectively. The samples were added to the loading buffer, and the mixtures were heated in boiling water for 10 min. The protein (15 μg) was transferred to each well, and the gels were stained with Coomassie Brilliant Blue G-250 after electrophoresis. Image analysis was performed using a gel scanner (Amersham Pharmacia Biotech, Uppsala, Sweden).

### Enzyme-linked immunosorbent assay (ELISA)

IgG-binding capacity was determined by ELISA, according to the method described by^25^, with minor modifications. A 96-well microtiter plate was coated with 100 µg/mL protein in phosphate-buffered saline (PBS) overnight at 4 °C. Then, the plate was washed three times with PBS containing 0.05% Tween 20 (PBST), followed by blocking with 3% gelatin in PBS for 1 h at 37 °C. The plate was washed, and incubated with serum from rabbits allergic to CM (100 μL; 1:1,000 in PBS) for 1 h at 37 °C. After washing, HRP-labeled goat anti-rabbit IgG antibody was added to the plate (100 μL; 1:5000 in PBS) for 1 h at 37 °C. After washing, OPD was added and incubated for 15 min at 37 °C in the dark. The reaction was stopped by adding 50 μL of 2 mol/L H_2_SO_4_. The absorbance was measured at 490 nm using an ELISA reader (BioRad, Hercules, CA).

### Determination of total protein content

The total protein content was measured using the Quick Start Bradford Assay kit (BioRad), in accordance with the manufacturer's instructions^26^. BSA was used as the standard.

### Determination of AA composition

**Figure 1:**
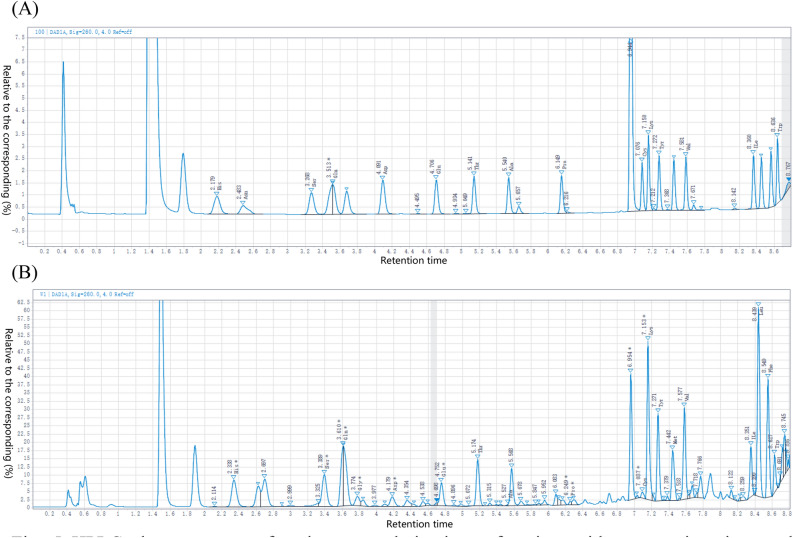
HPLC chromatogram of aminoenone derivatives of amino acids, ammonium ion, and biogenic amines at 280 nm. Standard solution (**A**); Representative sample (**B**). Met, Methionine; Val, Valine; ILE, Isoleucine; Leu, Leucine; Phe, Phenylalanine; Trp, Tryptophan; Lys, Lysine; Thr, Threonine; His, Histidine; Asn, Asparagine; Ser, Serine; Gln, Glutamine; Arg, Arginine; Gly, Glycine; Asp, Aspartic acid; Glu, Glutamic acid; Ala, Alanine; Pro, Proline; Cys, Cysteine; Tyr, Tyrosine.

AA composition analysis was performed using Agilent 1200 HPLC (Agilent Technologies, Madrid, Spain), according to the method of^27^. Chromatographic separation (Fig. 1) was performed using a Zorbax Eclipse XDB C18 column (5 mm) and an Agilent guard cartridge C18 (5 mm). Sample (50 µL) was added to the column and eluted at a flow rate of 0.9 mL min^−1^, according to the linear gradient used by^28^.

### Evaluation of sensory characteristics

The sensory properties of the samples were evaluated using an electronic tongue (ISENSO SuperTongue, USA), according to the method described by^29^. The sensory attributes of samples, including sourness, sweetness, bitterness, saltiness, umami, astringency, and richness were evaluated. Untreated CM samples were used as controls.

### Measurement of color

The color of the samples was determined with a chromameter (Minolta CM-3600d, Japan), which was calibrated using a standard white and black plate; L* represents lightness, ranging from black to white (0.00–100.00). In addition, a positive value of a* indicates red, and a negative number indicates green. A positive value of b* indicates yellow, and a negative value represents blue.

### Determination of flow behavior

The flow behavior of the samples was measured using a controlled stress rheometer (AR 2000, TA Instruments, New Castle, DE, USA) and a double concentric cylinder. The sample (3 mL) was added to the sample compartment, and the temperature was set to 25 °C during the measurement. The shear rate γ was set from 0.01 to 100 s^−1^ for 5 min. Later, a steady-state test was carried out to record the variation in viscosity and shear stress against the shear rate during the experiment.

### Statistical analysis

All experiments were performed in triplicate and the results are reported as the mean ± standard deviation of three independent assays. Statistical calculations were performed using GraphPad Prism 5.0 (GraphPad Software, San Diego, CA). The statistical differences were calculated by analysis of variance and the Duncan test using SPSS 22.0 software (SPSS Inc., USA). In all cases, *P* values < 0.05 were considered statistically significant.

## Results

### Degree of hydrolysis

**Figure 2:**
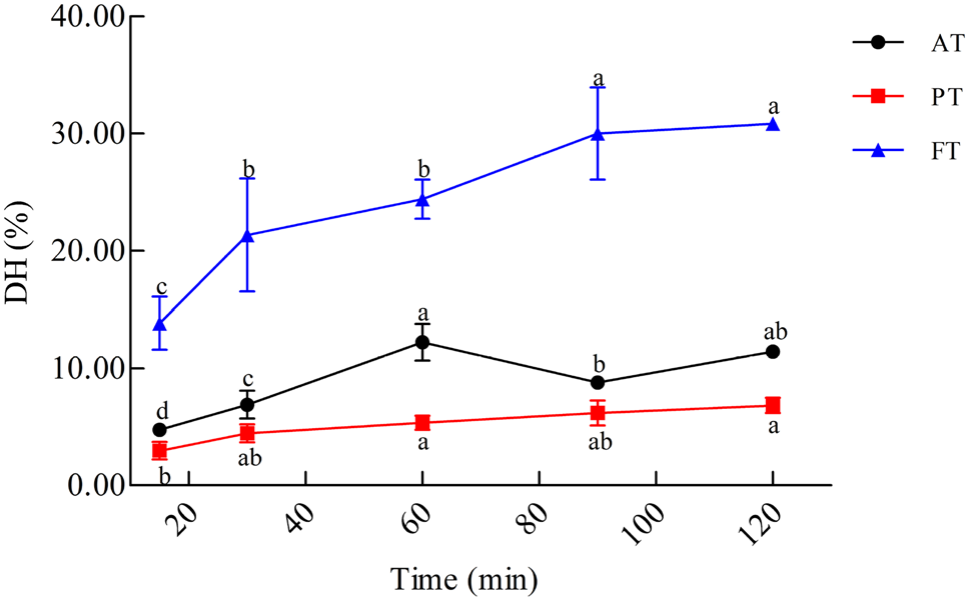
The degree of hydrolysis (DH) of CM with different enzymatic hydrolysis time. Alcalase-treated cow milk (AT), Protamex-treated cow milk (PT), Flavourzyme-treated cow milk (FT). Each value represents the mean of three independent experiments ± SD. Different letters indicate significant differences among groups (*P* < 0.05).

The DH of enzymatic hydrolysis-treated CM (HM) produced by Alcalase, Protamex, and Flavourzyme was quantified using the OPA method (Fig. 2). It can be seen that the DH of Flavourzyme-treated CM (FT) and Protamex-treated CM (PT) gradually increased with the time of enzymatic hydrolysis. Flavourzyme displayed the ability to hydrolyze CM, and the DH ranged from 13.87% to 31.36%. The hydrolysis capacity of Protamex to CM was lower, with the DH ranging from 2.99% to 6.86%. In addition, with the increase in enzymatic hydrolysis time, the DH of Alcalase-treated CM (AT) showed an increasing trend, and reached a maximum value (13.81%) after 60 min, but as the enzymatic hydrolysis time reached 90–120 min, the DH decreased gradually (90 min: 8.81%; 120 min: 11.43%).

### SDS-PAGE profile

**Figure 3:**
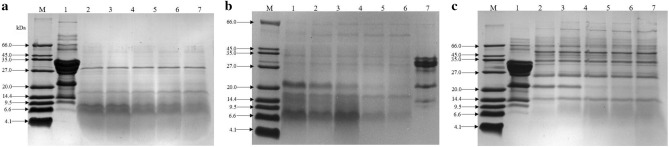
SDS-PAGE of CM with different enzymatic hydrolysis time. SDS-PAGE analysis. (**a**–**c**) represents Alcalase-treated cow milk (AT), Protamex-treated cow milk (PT), Flavourzyme-treated cow milk (FT), respectively. (**a**, **c**) Lane 1: marker; lane 2: CM; lane 3: 15 min; lane 4: 30 min; lane 5: 60 min; lane 6: 90 min; lane 7: 120 min; (**b**) Lane 1: marker; lane 2: 15 min; lane 3: 30 min; lane 4: 60 min; lane 5: 90 min; lane 6: 120 min; lane 7: CM;

Figure 3 shows the electrophoretic patterns of CM and HM obtained with Alcalase, Protamex, and Flavourzyme. The electrophoretic patterns of CM showed protein bands with apparent molecular weights (MWs) that ranged from to 4.0 to 66.1 kDa. The electrophoretic pattern exhibited three higher intensity bands with apparent MWs of approximately 19.0–25.0, 18.0, and 14.0 kDa, which most likely correspond to CNs, BLG, and ALA, respectively, and also showed a lower density protein band that might correspond to BSA (66.1 kDa). However, the electrophoretic patterns of HM showed that the protein bands for CN, BLG, and ALA became less visible. Among these, the protein bands for CN, BLG, and ALA in AT were not significant changed with increasing time. As for FT, the band of BLG vanished when the enzymatic hydrolysis time was 60 min. Notably, as the enzymatic hydrolysis time reached 90 min, the bands (CNs, BLG, and ALA) of PT vanished completely.

### IgG-binding capacity

**Figure 4:**
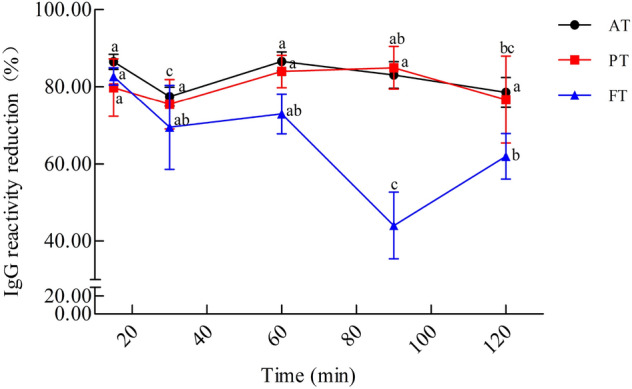
IgG reactivity of CM hydrolyzed with different enzymatic hydrolysis time. Alcalase-treated cow milk (AT), Protamex-treated cow milk (PT), Flavourzyme-treated cow milk (FT). Each value represents the mean of three independent experiments ± SD. Different letters indicate significant differences among groups (*P* < 0.05).

The IgG-binding capacity of HM was evaluated by icELISA using anti-CM rabbit polyclonal antibodies (Fig. 4). The IgG reactivity reduction of HM ranged from 44.05% to 86.55%. With increasing time of enzymatic hydrolysis, the reduction in IgG reactivity of AT and PT did not decrease significantly *(P* > 0.05). Interestingly, the enzymatic hydrolysis time reached 15–60 min, the reduction in IgG reactivity of FT was 82.27%, 69.46%, and 72.94%, but when the enzymatic hydrolysis time reached 90 min, the IgG reactivity reduction of FT significantly increased (44.05%).

### Total protein content

**Figure 5:**
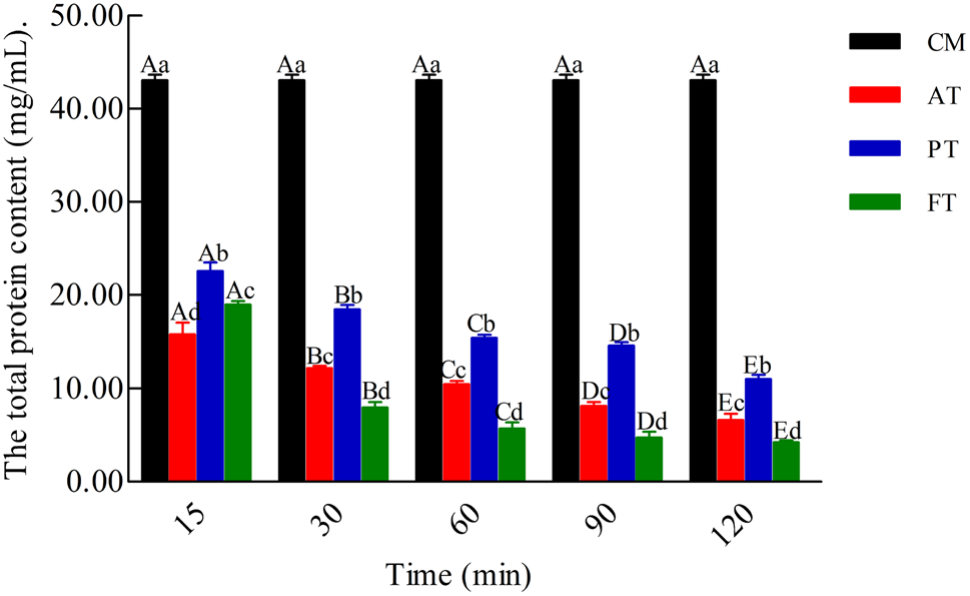
Effect of different enzymatic hydrolysis on the total protein of cow milk (mg/mL). Alcalase-treated cow milk (AT), Protamex-treated cow milk (PT), Flavourzyme-treated cow milk (FT). Each value represents the mean of three independent experiments ± SD. Different letters indicate significant differences among groups (*P* < 0.05).

Figure 5 shows that with the increase in enzymatic hydrolysis time, the total protein content was significantly reduced (*P* < 0.05). Among these, the content of FT was significantly lower than that of AT and PT (*P* < 0.05).

### AA composition

**Figure 6:**
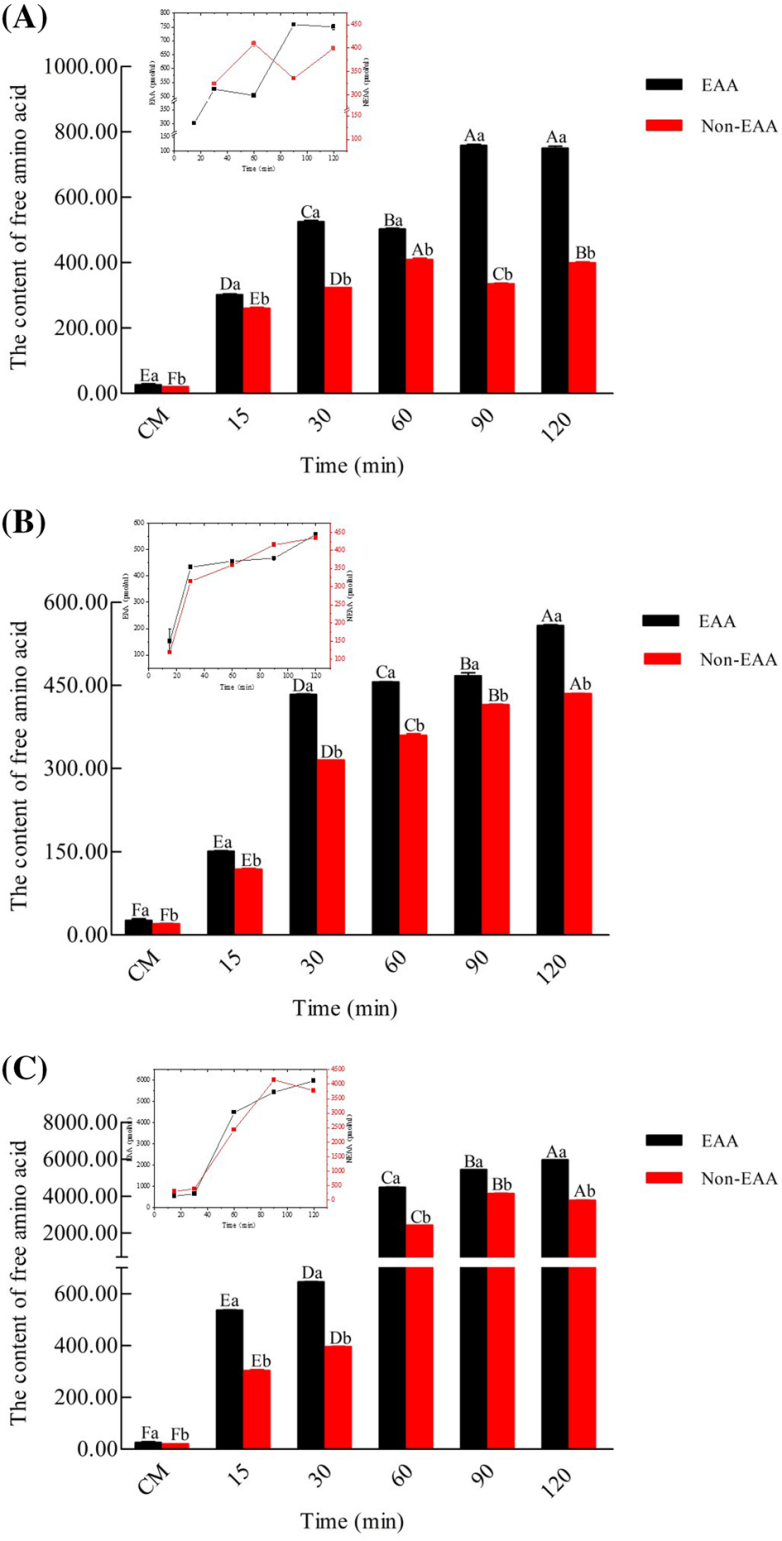
The content of amino acids of CM, AT (**A**), PT (**B**) and FT (**C**). Alcalase-treated cow milk (AT), Protamex-treated cow milk (PT), Flavourzyme-treated cow milk (FT). Essential amino acids (EAAs); Non-essential amino acids (Non-EAAs).

The AA composition was analyzed using HPLC. The total free amino acid (TFAA) of HM significantly increased (*P* < 0.05) in comparison with CM (Fig. 6). The content of essential amino acids (EAAs) and non-essential amino acids (non-EAAs) significantly increased with time. Furthermore, the content of TFAAs in FT was higher than those of AT and PT. With respect to the AT, when the enzymatic hydrolysis time reached 120 min, there was a significant increase in the content of Thr, Tyr, Glu, Asp, Ile, and Leu, with levels approximately 104.79-fold (Thr), 49.85-fold (Tyr), 43.63-fold (Glu), 43.59-fold (Asp), 38.60-fold (Ile), and 37.40-fold (Leu) higher than that in CM. The PT levels were approximately 96.69-fold (Ile), 56.45-fold (Glu), 39.43-fold (Thr), 33.09-fold (33.09), and 29.58 (Tyr) higher than that of CM. There was a significant increase in the content of His, Thr, Tyr, Phe, Leu, and Glu for FT, with the levels approximately 1344.77-fold (His), 605.45-fold (Thr), 520. 06-fold (Tyr), 511.41-fold (Phe), 285.03-fold (Leu), and 151.48-fold (Glu) higher than that in CM.

**Figure 7:**
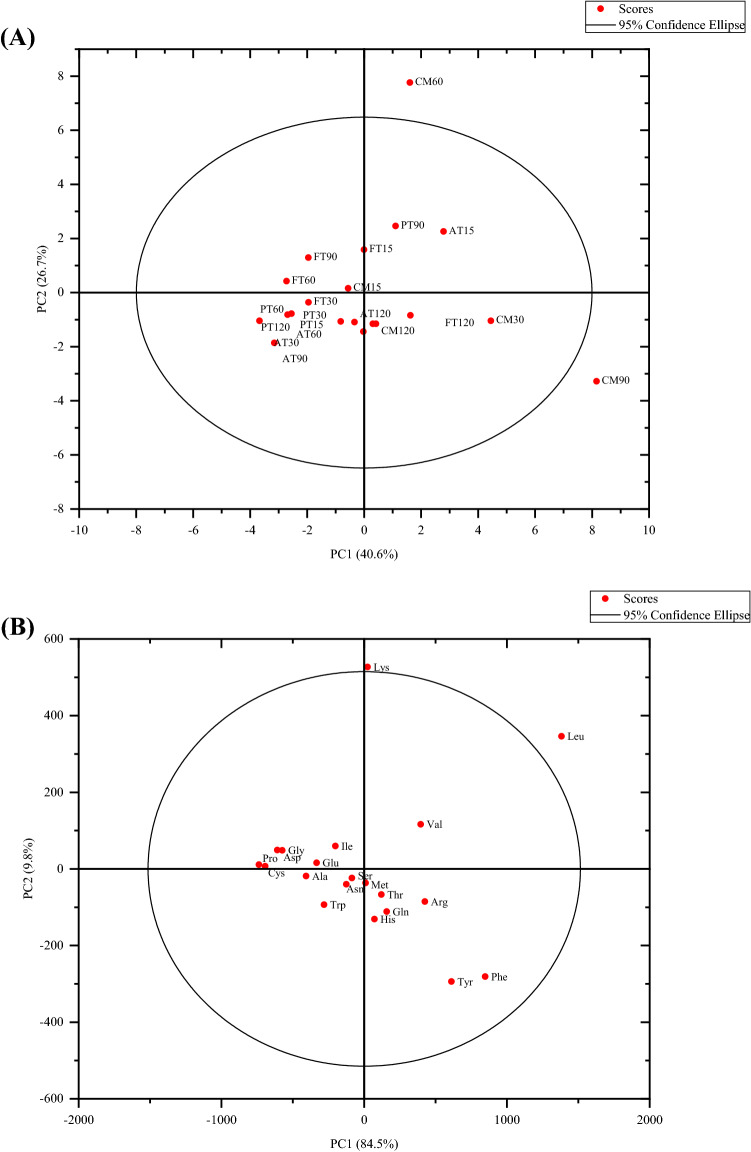
PCA score plot (**A**) and PCA correlation loadings plot (**B**) of the principal components of free amino acids from CM, AT, PT and FT. Alcalase-treated cow milk (AT), Protamex-treated cow milk (PT), Flavourzyme-treated cow milk (FT). Each dot represents identified and quantified free amino acids.

**Figure 8:**
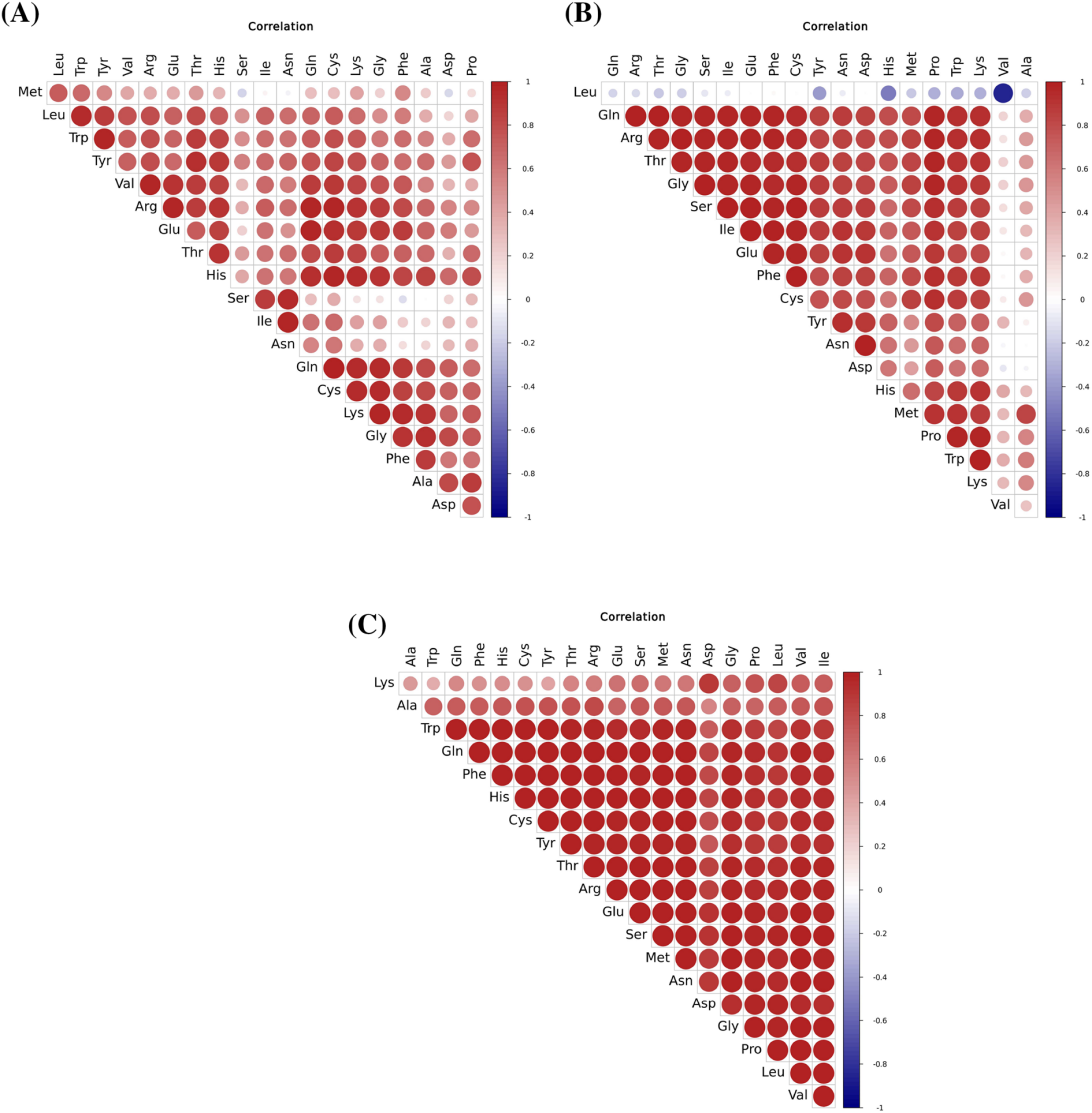
Correlation analysis of amino acids in AT (**A**), PT (**B**) and FT (**C**). Alcalase-treated cow milk (AT), Protamex-treated cow milk (PT), Flavourzyme-treated cow milk (FT).

**Figure 9:**
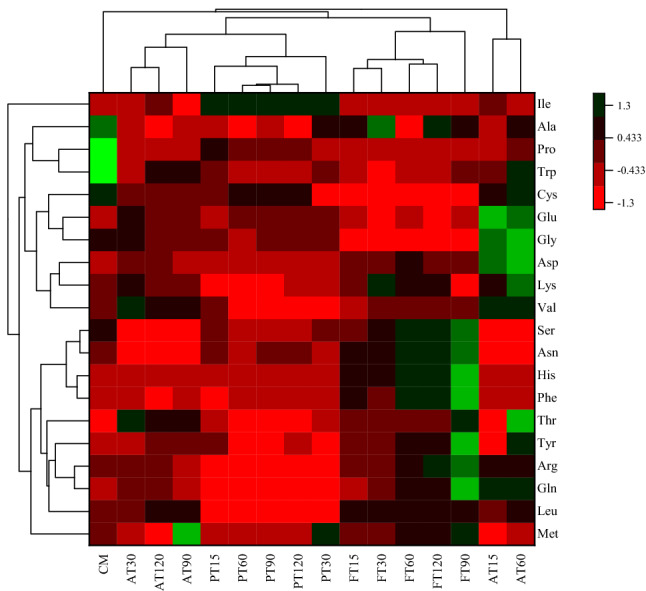
Hierarchical clustering of free amino acids in CM, AT, PT and FT (*P* < 0.05). The bar color represents a logarithmic scale from − 1.3 to 1.3. Alcalase-treated cow milk (AT), Protamex-treated cow milk (PT), Flavourzyme-treated cow milk (FT). Each column indicates a sample, and each row indicates a free amino acid.

PCA of EAAs and non-EAAs showed that two principal components, the cumulative explained variance, was 40.6%, indicating that these two principal components could not be distinguished between CM and HM (Fig. 7). PC1 mainly contained Met, Val, Leu, Phe, Thr, His, Gln, Arg, and Tyr. PC2 principally included Ile, Leu, Lys, Gly, Asp, Glu, Pro, and Cys. Figure 8 shows the Pearson correlations between the 20 AAs in the CM and HM. Of these correlations, 195 were positive and 5 were negative (AT), 177 were positive and 23 were negative (PT), and 200 were positive (FT). In the correlations of AT, Cys and Gln had the largest positive autocorrelation (0.98474), Ser and Met had the lowest negative autocorrelation (-0.16879); for PT, Ile and Glu had the largest positive autocorrelation (0.99515), Val and Leu had the lowest negative autocorrelation (-0.85023). As for FT, Asn and Met had the largest positive autocorrelation (0.99929), Trp and Lys had the lowest negative autocorrelation (0.37417). In order to compare the expression patterns of AAs from CM and HM, hierarchical cluster analysis was used to analyze the significant, differentially expressed AAs (Fig. 9).

### Taste

**Figure 10:**
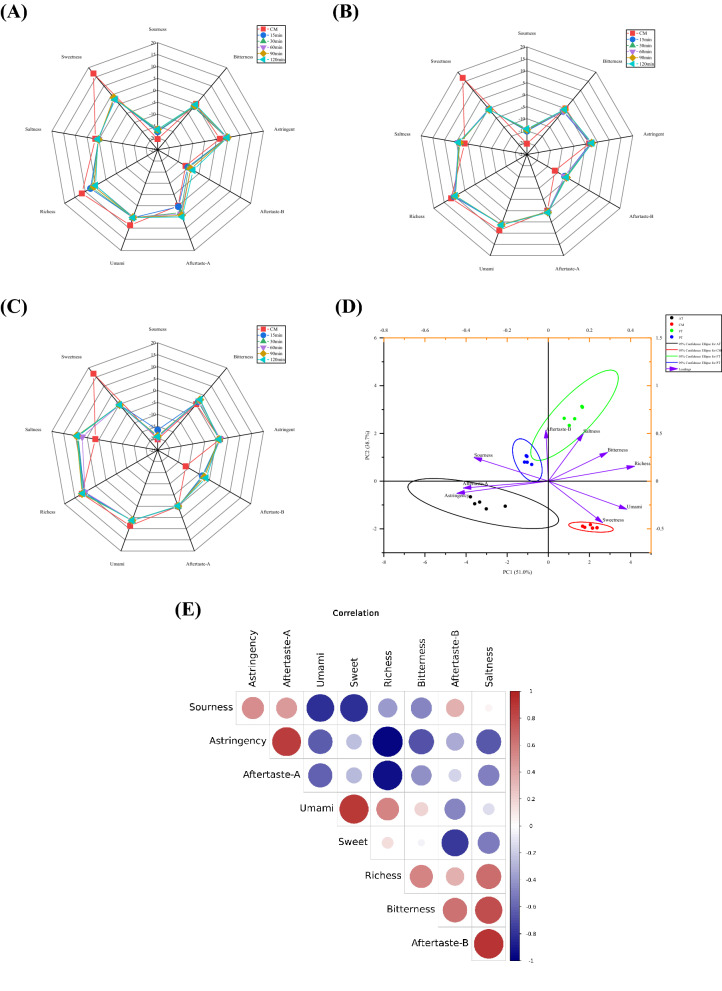
Radar distribution of taste attributes of AT (**A**), PT (**B**), FT (**C**), PCA score plot (**D**) and correlation analysis of sensory (**E**). Alcalase-treated cow milk (AT), Protamex-treated cow milk (PT), Flavourzyme-treated cow milk (FT).

The sensory characteristics of CM and HM were determined using an electronic tongue (e-tongue). High intensity positive tastes, such as sweetness, richness, and umami, were found in CM (Fig. 10). Negative tastes, such as sourness, an aftertaste of bitterness (aftertaste-B), and astringency were observed in HM, showing an increasing trend over time. In particular, the sweetness of AT, PT, and FT significantly decreased in comparison with CM (*P* < 0.05). Figure 10D shows that AT, PT, FT, and CM can be clearly distinguished in the PCA score plot. FT showed saltiness and bitterness, and PT had a slight sourness, and AT had heavy astringency and an aftertaste of astringency (aftertaste-A). Additionally, Fig. 10E shows a significant correlation between the sensory characteristics of AT, PT, FT, and CM. Among these correlations, 15 were positive and the remaining 21 were negative. The largest positive autocorrelation was observed between saltiness and aftertaste-B (0.91340), whereas the lowest negative autocorrelation was found between richness and astringency (-0.96508).

### Color

**Figure 11:**
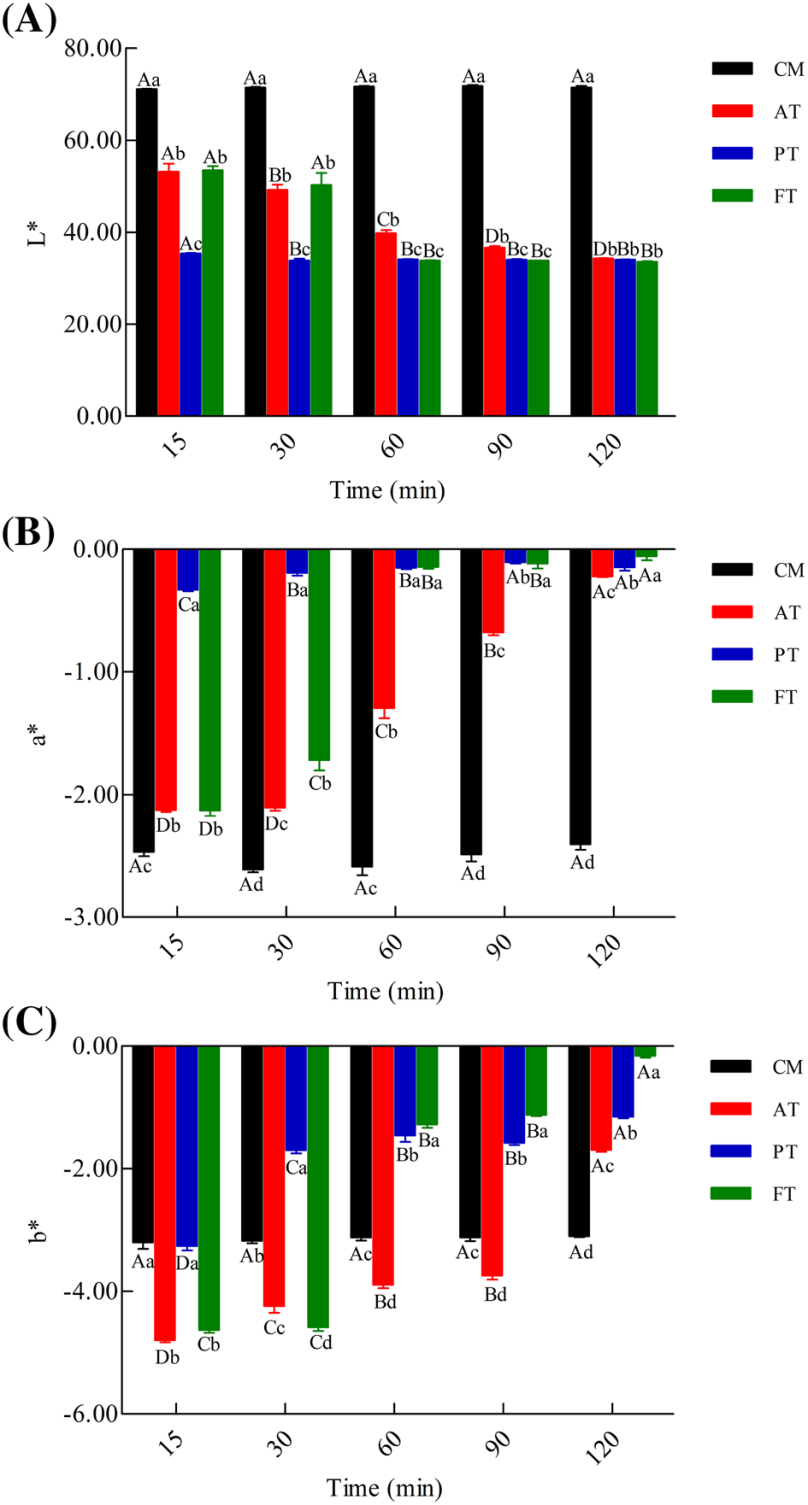
Color measurement L* (**A**), a* (**B**), b*(**C**) of CM, AT, PT and FT. Alcalase-treated cow milk (AT), Protamex-treated cow milk (PT), Flavourzyme-treated cow milk (FT).

The results of L*, a*, and b* values of CM, AT, PT, and FT are shown in Fig. 11. CM had a high L*(71.94) and low a* (− 2.50) and b*(− 3.02), and the colors of AT, PT, and FT were distinctly different from that of CM. In addition, AT, PT, and FT had a significant decrease in L*, and a significant increase in a* and b* at different enzymatic hydrolysis times. Interestingly, as enzymatic hydrolysis time reached 120 min, the b* of AT, PT, and FT reached minimum values (− 1.686, − 1.146, and − 0.156, respectively). PT (90 min), AT, and FT (all 120 min) achieved maximum values (− 0.22, − 0.11, and − 0.06, respectively) in a*; in contrast, their L* values were 34.35, 34.10, and 33.65, respectively, which were the lowest values obtained.

### Flow behavior

**Figure 12:**
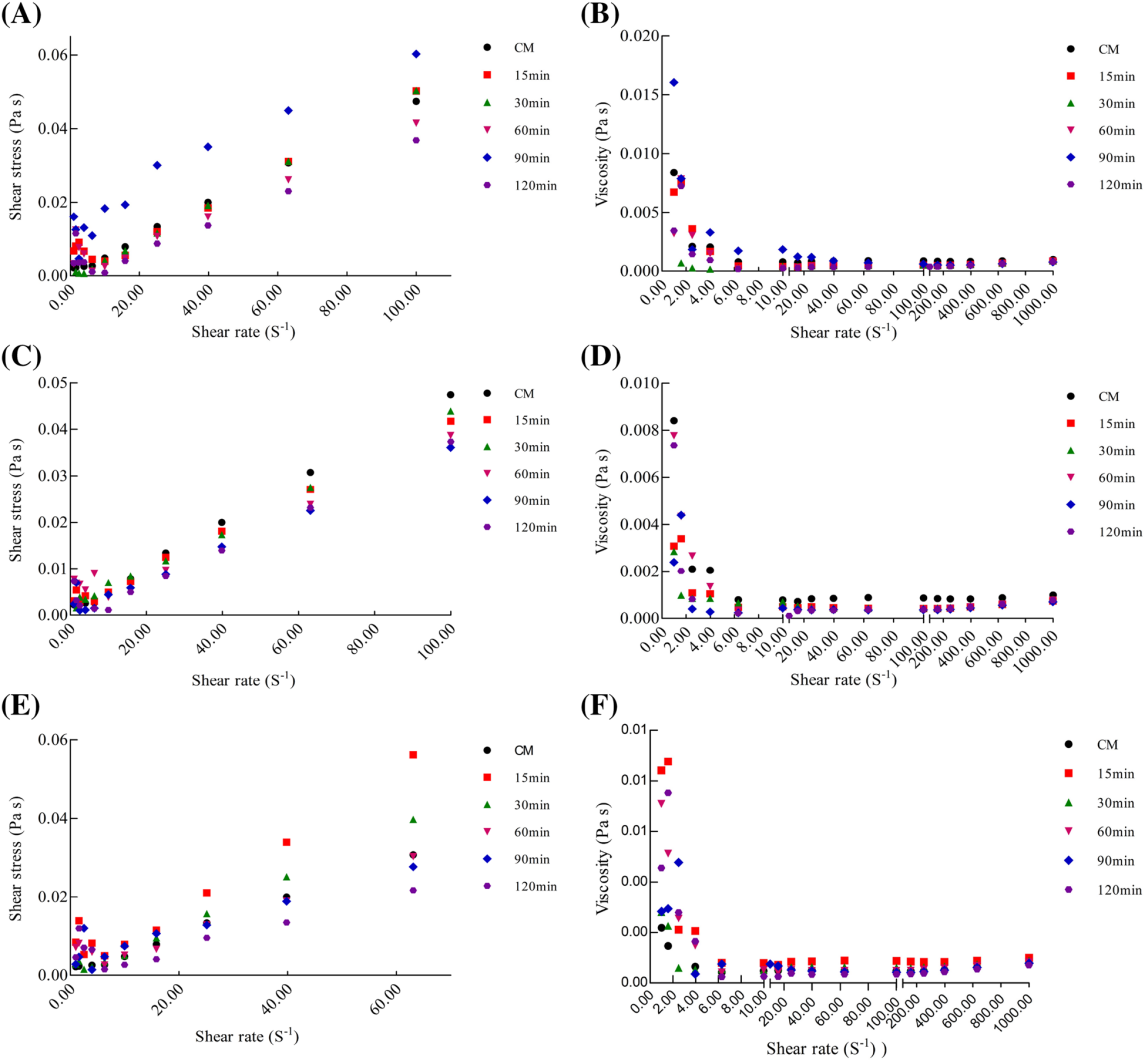
The shear stress and viscosity of CM, AT (**A**, **B**), PT (**C**, **D**) and FT (**E**, **F**). Alcalase-treated cow milk (AT), Protamex-treated cow milk (PT), Flavourzyme-treated cow milk (FT).

The relationship between shear rate and shear stress or apparent viscosity of CM, AT, PT, and FT were assessed (Fig. 12). The trend of the relationship between shear rate and apparent viscosity was similar among CM and AT, PT, or FT. Generally, apparent viscosity decreased with the increasing intensity of shear rate, and the apparent viscosity gradually stabilized when the shear rate reached a high level (0.10–10.00 S^−1^), which exhibited pseudoplastic fluid behavior. At a lower shear rate (0.01–0.10 S^−1^) the apparent viscosity decreased sharply and the shear thinning effects were observed.

Furthermore, the shear stress significantly increased with the intensity reinforcement of the shear rate (Fig. 12A,C,E). Notably, when CM was incubated with Alcalase, Protamex, and Flavourzyme, and enzymatic hydrolysis time was 90 min, 60 min, and 15 min, respectively, and the apparent viscosity and shear stress reached high levels.

## Discussion

Many strategies have been widely used to improve the functional properties of food, including chemical and enzymatic modifications, but enzymatic hydrolysis might be the preferable method due to milder process conditions, higher specificity, and minimal by-product generation during the reaction^30^. Moreover, Wróblewska et al. suggested that enzymatic hydrolysis is the most effective method to alter the immunoreactivity of food proteins^31^. In addition, Yang et al. demonstrated that enzymatic hydrolysis is a safe and mild process method, which can break peptide and disulfide bonds and result in the collapse of conformational and linear epitopes, destroying the allergenic proteins. Most importantly, it does not reduce the nutritional quality or destroy the nutritional composition^32^. In the present study, we found that CM proteins were more sensitive to Flavourzyme and exhibited a higher DH. While Alcalase and Protamex showed a lower DH than Flavourzyme, the DH did not increase with time. Similarly, some studies have indicated that limited hydrolysis, ranging from 1.0 to 15.0%, was needed, which was beneficial for improving the functional properties of food^33^.

Enzymatic hydrolysis is the process of using certain digestive enzymes to break down the peptide bonds of proteins and convert the whole protein into smaller peptide fragments. However, the species of enzymes are different, and the degradation abilities of these enzymes also vary^34^. Our findings showed that the electrophoretic patterns of HM became less visible with increasing time, especially the bands of PT, which vanished completely with increasing enzymatic hydrolysis time. Some studies have reported that the protein bands of AT became less visible, compared to WPC, and a large amount of high MW proteins decreased, with many peptide bands below 14.0 kDa^35^. Previous studies have demonstrated that WPC obtained from Alcalase (0.6 L) generates lower MW peptides, ranging from 10.0–20.0 kDa^36^.

During the enzymatic hydrolysis reaction, the structure of food allergens is altered, which interferes with the antigen–antibody complex-forming ability. In the present study, we found that the IgG-binding capacity of HM was significantly reduced. Thus, we speculated that this might be due to the splitting of epitope sequences during enzymatic hydrolysis. Similar research carried out by Jordan et al. suggested that enzymatic hydrolysis could damage or destroy antigenic epitopes of allergenic proteins, resulting in the reduction of IgG-binding capacity and immunoreactivity^37^. In addition, Villas-Boas et al. indicated that enzymatic hydrolysis reduced the amount of allergenic epitopes, and the IgE or IgG-binding ability of β-LG could be significantly decreased^38^. Jing et al. showed that reduction of the IgE-binding ability of ALA and BLG in CM was reduced, ranging from 15.0 to 90.0%^39^. These findings are in line with the DH of CM obtained with Alcalase, Protamex, and Flavourzyme, indicating that enzymatic hydrolysis is an effective method to reduce the immunoreactivity of CM. Taken together, enzymatic hydrolysis could effectively reduce the immunoreactivity of CM, but the effect of enzymatic hydrolysis on the nutritional properties of natural CM should be further studied.

The contents of the total protein and AA composition were systematically analyzed in our study. Our findings showed that the total protein content was significantly lower than that of CM, and the content of FT was significantly lower than that of AT and PT. These results are consistent with the DH of AT, PT, and FT. The reason might be that Flavourzyme was obtained by the controlled fermentation of *Aspergillus oryzae*, which is a mixture of proteases with both exo- and endo-peptidase activities, which can accelerate protein hydrolysis^40^. Some studies have demonstrated that Alcalase mainly catalyzes the amide bond formed by the carboxyl group of hydrophobic AAs, hydrolyzes the peptide bonds inside the protein, and generates polypeptides with relatively small MWs^41^.

AAs are important nutrients in the body, and most people in developed countries get enough AAs from proteins in the diet^42^. The composition of EAAs and non-EAAs were evaluated in this study, and it was found that the TFAAs of HM significantly increased, and the content of TFAAs in FT was higher than those of AT and PT. Previous studies have suggested that the AAs of hydrolysates depend on the manufacturing technology, such as the type of enzymes, enzymatic conditions, and the extent of proteolysis^43^. Yvon & Rijnen evaluated the hydrolysates of cheese, and found that the content of EAAs and non-EAAs varied significantly according to the extent of proteolysis^44^. In particular, we found that the Thr, Leu, Ile, Trp, His, Glu, and Tyr in HM were significantly higher than those in CM. Liang et al. reported that FAAs could be directly absorbed by the human body, which helps the body regulate the immune system and provide energy for the body and brain^45^. Baldeón et al. observed that the content, composition, and ratio of FAAs could reflect the nutritional value of food products^46^. In addition, recent studies have indicated that Trp can promote the growth and development of infants and children, enhance intelligence, and even play a significant role in the treatment of diseases such as cancer^47^. Zhang et al. suggested that His is very important for infant growth and development, which can promote the early development of immune system function of infants, strengthen physiological metabolism, stabilize the utilization rhythm of proteins in the body, and promote body development in infants^48^. Therefore, our findings provide a theoretical foundation for the development of functional and hypoallergenic dairy products.

Taste is an important characteristic of CM products, with a good taste being a key factor in gaining the favor of consumers. Thus, the effect of Alcalase, Protamex, and Flavourzyme treatment on the taste of CM was evaluated in this study. Our results showed that sourness, aftertaste-B, and astringency were observed in HM, and the sweetness significantly decreased, which indicated that enzymatic hydrolysis with Alcalase, Protamex, and Flavourzyme significantly affected the taste profile of CM. This may be due to the break-down of proteins, which results in the release of a large amount of FAAs and small-molecule peptides with different taste characteristics^49^. Similarly, Phat et al. reported that the different taste of peanut meal hydrolysates might be due to different compositions of FAAs and polypeptides^50^.

Here, color parameters indicated that the color of HM is significantly different to that of CM (lower L*, and higher b* and a* values). This phenomenon occurred because the protein micelles changed into smaller particles during enzymatic hydrolysis. Furthermore, the change in color could be beneficial for incorporation into certain foods, such as cookies and extruded snacks^51^. A similar result was obtained by^52^, who indicated that a significant decrease in L* values were observed in camel milk treated with ultra-high pressure, which is in line with our findings.

Flow behavior can be used to describe and measure the texture of products; it can not only accurately reflect the essential properties of food, but also guide the development of CM products. The findings of the present study showed that the relationship between the shear rate and apparent viscosity of HM is similar to that of CM, and with the increase in shear rate intensity, the apparent viscosity decreased, while shear stress significantly increased. Falcone et al. suggested that the change in apparent viscosity and shear stress is closely related to the shear rate, and the change in apparent viscosity and shear stress might be caused by changes in the shape of CN and other dispersion system particles during enzymatic hydrolysis^53^. Deshpande et al. also reported that the viscosity of dairy products changed because the shear stress destroyed the non-covalent interaction that formed the aggregates^54^.

In conclusion, our findings indicated that enzymatic hydrolysis treatment with Alcalase, Protamex, and Flavourzyme destroyed allergenic proteins, effectively reduced the immunoreactivity of CM, and increased the FAA content and nutritional quality. In particular, the antigenicity of CM was significantly reduced from 44.05% to 86.55%. Simultaneously, the taste, color, and flow behavior of CM were altered, the sweetness and richness intensity of HM significantly decreased, and astringency and bitterness were produced. A slight darker and more yellow color was observed in CM hydrolysate. In addition, apparent viscosity decreased and shear stress significantly increased with increasing shear rate intensity. The results will provide a solid theoretical foundation for the development of high-quality hypoallergenic dairy products.
